# Some Further Results on the Fractional Cumulative Entropy

**DOI:** 10.3390/e24081037

**Published:** 2022-07-28

**Authors:** Mohamed Kayid, Mansour Shrahili

**Affiliations:** Department of Statistics and Operations Research, College of Science, King Saud University, P.O. Box 2455, Riyadh 11451, Saudi Arabia; drkayid@ksu.edu.sa

**Keywords:** fractional cumulative entropy, fractional cumulative residual entropy, maximum order statistic, Shannon entropy, risk-adjusted premium

## Abstract

In this paper, the fractional cumulative entropy is considered to get its further properties and also its developments to dynamic cases. The measure is used to characterize a family of symmetric distributions and also another location family of distributions. The links between the fractional cumulative entropy and the classical differential entropy and some reliability quantities are also unveiled. In addition, the connection the measure has with the standard deviation is also found. We provide some examples to establish the variability property of this measure.

## 1. Introduction

The uncertainty in statistical information theory is an aspect of randomness which is measured by entropy, originally introduced by Shannon [1] in their seminal work. The entropy has been firstly proposed to quantify the uncertainty in a discrete random variable (RV). There are many generalizations of Shannon entropy recognized in the literature, defined by appending additional parameters. These generalizations make the entropies sensitive to different probability distributions (see Renyi [2] and Tsallis [3], among others). One such generalization is the fractional entropy (FE), which is based on fractional calculus. Moreover, the FE is a concave positive function with non-additivity property. From a physical point of view, there are also some descriptions associated with the Lesche and thermodynamic stability.

The Shannon entropy can be developed from the discrete to continuous case as below:

(1) $$H\left(X\right)=-\int_{0}^{\infty }f\left(x\right)\log f\left(x\right)dx,$$

where f(x) is the probability density function (PDF) of a nonnegative RV *X* with an absolutely continuous cumulative distribution function (CDF) F(x). Despite the many advantages of the differential entropy on the one hand, it has some disadvantages on the other hand. For example, it is very complicated to estimate the differential entropy of a continuous RV in terms of the empirical distribution arisen from samples. However, since the PDF exists, we can use this possibility. Furthermore, it can take negative values in calculations (cf. Rao et al. [4] and Rao [5]). For some recent work surveyed in the literature, we refer the reader to Kharazmi and Balakrishnan [6], Mohamed et al. [7], and the references therein.

An alternative and more direct quantity proposed by Rao et al. [4], using the survival function (SF) $\bar{F}\left(x\right)=1-F\left(x\right)$ in place of the PDF f(x), is given by:

(2) $$E\left(X\right)=-\int_{0}^{\infty }\bar{F}\left(x\right)\log\bar{F}\left(x\right)dx.$$

Properties of (2) and its dynamic version and some other generalization of this measure together with their properties are discussed in detail in Asadi and Zohrevand [8], Navarro et al. [9], Toomaj et al. [10], Psarrakos and Navarro [11], Psarrakos and Toomaj [12], Tahmasebi and Mohammadi [13], and Mohamed et al. [14], among others. By analogy with (2), Di Crescenzo and Longobardi [15] introduced cumulative entropy (CE) by substituting F(x) in place of PDF f(x), as:

(3) $$\mathrm{CE}\left(X\right)=-\int_{0}^{\infty }F\left(x\right)\log F\left(x\right)dx=\int_{0}^{\infty }F\left(x\right)T\left(x\right)dx,$$

where:

(4) $$T\left(x\right)=\int_{x}^{+\infty }\tau \left(u\right)du=-\log(F(x)),\;x>0,$$

is the cumulative reversed hazard rate (CRHR) function and τ(u)=f(u)/F(u),u>0, is the reversed hazard rate (RHR) function (see, e.g., Hanagal and Pandey [16]). Note that the integral in (4) is convergent for all x>0; however, T(0)=+∞. It is also evident that T(+∞)=0. It is obvious that 0≤CE(X)≤+∞, since the argument of the logarithm is a probability. Moreover, the CE becomes zero if, and only if *X* is a degenerate random variable. Properties of the cumulative entropy in the two-dimensional case have been argued in Ahmadi et al. [17]. For further properties of cumulative entropies, we refer the reader to Di Crescenzo and Longobardi [15,18], Kundu et al. [19], and references therein. The CE has been developed to more general cases, see, for instance, Di Crescenzo and Toomaj [20], Kayal [21], Kayal and Moharana [22], and references therein.

Recently, Xiong et al. [23] introduced the fractional cumulative residual entropy (FCRE) following the properties of the CRE in (2) and the FE as:

(5) $$E_{q}\left(X\right)=\int_{0}^{\infty }\bar{F}\left(x\right){\left[-\log\bar{F}\left(x\right)\right]}^{q}dx,$$

for all 0<q≤1. Xiong et al. [23] investigated several properties of this measure, including the effect of linear transformations of RVs on it. To be more specific, they showed that $E_{q}\left(aX+b\right)=aE_{q}\left(X\right)$, where a>0 and b≥0. They also obtained several bounds and used the concept of stochastic orders to establish some insightful comparison of distributions following their corresponding FCREs.

This article concentrates on a newly proposed measure of uncertainty that complements the FCRE from (5). This measure has been derived by transplantation of the cumulative distribution function with the survival function in (5) (see Di Crescenzo et al. [24]). This measure extends the cumulative entropy (3) to a fractional order. In fact, our definition is a special case, but equivalent in the conception, of that given in Di Crescenzo et al. [24] given by:

(6) $$CE_{q}\left(X\right)=\frac{1}{\Gamma (q+1)}\int_{0}^{\infty }F\left(x\right){\left[T\left(x\right)\right]}^{q}dx,\;q>0.$$

We give an overview of the paper: the fractional cumulative entropy is first described in Section 2. For generic and linear transformations of RVs, the measure is developed and some stochastic orderings are considered. We utilize some well-known stochastic orderings. Some instructive example are given to illustrate the concepts. In Section 3, we present several bounds and establish some inequalities involving the FCE. We provide several characterization results of symmetric distributions in Section 4. In Section 5, we finally conclude the paper with some remarks and illustrations about our future plan.

## 2. Fractional Cumulative Entropy

Throughout the paper, we assume that *X* is a nonnegative RV with an absolutely continuous CDF F(x). In analogy with (5), we define the fractional cumulative entropy (FCE) of *X* as follows:

(7) $${\mathrm{CE}}_{q}\left(X\right)=\int_{0}^{\infty }F\left(x\right){\left[-\log F\left(x\right)\right]}^{q}dx=\int_{0}^{\infty }F\left(x\right){\left[T\left(x\right)\right]}^{q}dx,$$

for all 0<q≤1. It is clear that $E_{0}\left(X\right)=\int_{0}^{\infty }F\left(x\right)dx,$ which may be divergent.

We remark that our definition is a special case of the definition given by Di Crescenzo et al. [24] in (6). Therefore, in this case, the fractional cumulative entropy is given by:

$${\mathrm{CE}}_{q}\left(X\right)=\Gamma \left(q+1\right)CE_{q}\left(X\right),\;0<q\leq 1.$$

Moreover, FCE becomes the CE when the parameter *q* takes the value one, i.e., ${\mathrm{CE}}_{1}\left(X\right)=\mathrm{CE}\left(X\right)$, as given in (3). On the other hand, the FCE is nonnegative and a concave function of the distribution, but it is a convex function of the parameter q. From (7), it is clear that ${\mathrm{CE}}_{q}\left(X\right)$ takes values in [0,∞]. In particular, ${\mathrm{CE}}_{q}\left(X\right)=0$ for all 0<q≤1 if, and only if *X* is a constant.

**Remark** **1.** *Let us assume an RV X with finite mean μ=E(X)<+∞. The FCE ${\mathrm{CE}}_{q}\left(X\right)$ is equal with the fractional cumulative residual entropy (FCRE) $E_{q}\left(X\right)$ if the distribution of X is symmetric around μ, i.e., if F(μ+x)=1−F(μ−x) for all x>0.*

The next example makes the comparison of ${\mathrm{CE}}_{q}\left(X\right)$ for 0<q≤1 with the standard deviation σ(X) for a number of reputable distributions tabulated by Di Crescenzo et al. [24].

**Example** **1.**  *(a) Assume that X is distributed uniformly in [0,b], for b>0, which has the standard deviation:*

$$\sigma \left(X\right)=\frac{b}{2\sqrt{3}}.$$

*The straightforward computation yields:*

(8) $${\mathrm{CE}}_{q}\left(X\right)=\frac{b\Gamma (q+1)}{2^{q+1}},\;0<q\leq 1.$$

*In this case, $E_{q}\left(X\right)={\mathrm{CE}}_{q}\left(X\right)$ due to Remark 1. Moreover, we have:*

$${\mathrm{CE}}_{q}\left(X\right)=\frac{\sqrt{3}\Gamma \left(q+1\right)}{2^{q}}\sigma \left(X\right).$$

*Thus, we have ${\mathrm{CE}}_{q}\left(X\right)>\sigma \left(X\right)$ for all 0<q<0.6378127 and ${\mathrm{CE}}_{q}\left(X\right)<\sigma \left(X\right)$ for all 0.6378127≤q≤1.*

*(b) Let X have the Fréchet distribution with the CDF $F\left(x\right)=e^{-\frac{a}{x^{5}}},\;x>0$ with a>0, with the standard deviation:*

$$\sigma \left(X\right)=\sqrt[{5}]{a}\sqrt{\Gamma \left(1-\frac{2}{a}\right)-\Gamma^{2}\left(1-\frac{1}{a}\right)},$$

*for a>2. Recalling (7), we obtain:*

$${\mathrm{CE}}_{q}\left(X\right)=\frac{\sqrt[{5}]{a}}{5}\Gamma \left(q-\frac{1}{5}\right),$$

*for all 0.2<q≤1. Thus, we have:*

(9) $${\mathrm{CE}}_{q}\left(X\right)=s_{q}\left(a\right)\sigma \left(X\right),$$

*where:*

$$s_{q}\left(a\right)=\frac{\Gamma \left(q-\frac{1}{5}\right)}{5\sqrt{\Gamma \left(1-\frac{2}{a}\right)-\Gamma^{2}\left(1-\frac{1}{a}\right)}},\;\mathrm{for}\;\mathrm{all}\;0.2<q\leq 1.$$

**Figure 1:**
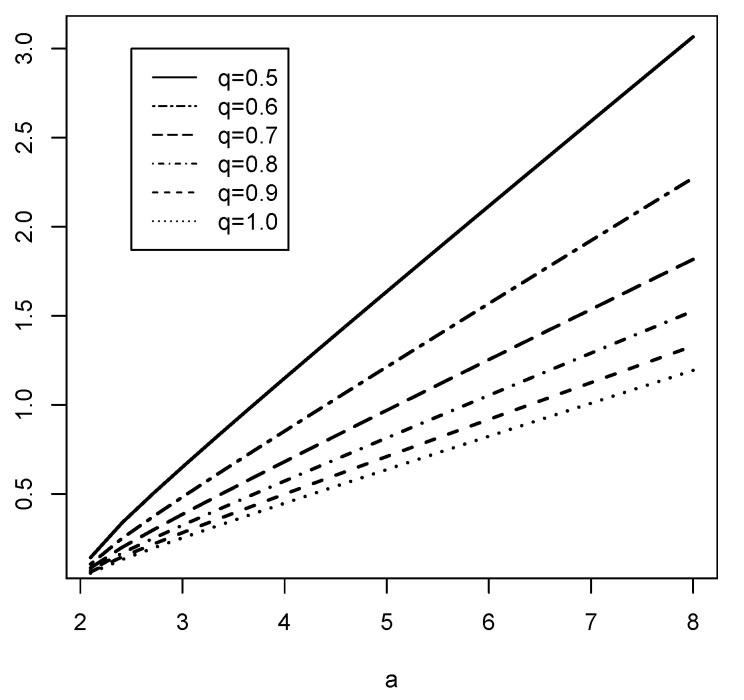
The functions $s_{q}\left(a\right)$ of Fr*é*chet distribution for different values of 0.2<q≤1 and a>2.

*Comparing the functions $s_{q}\left(a\right)$ to 1 as shown in Figure 1, and considering (9), we have the following results given in Table 1. One can see for a>2 that there exists a number $a_{0}\in \left(2,\infty \right)$ for which ${\mathrm{CE}}_{q}\left(X\right)>\left(<\right)\sigma \left(X\right)$ for $a>\left(<\right)a_{0}$ for all 0.2<q≤1.*

Note that ${\mathrm{CE}}_{q}\left(X\right)={\mathrm{CE}}_{q}\left(Y\right),\;0<q\leq 1$ does not imply that *X* is equal in distribution with *Y*; however, the converse holds. Let us now discuss the effect of an increasing transformation on the FCE. We omit the proof, as it is straightforward.

**Lemma** **1.** *If Y=ϕ(X), where ϕ(·) is a strictly increasing and differentiable function, then:*

(10) $${\mathrm{CE}}_{q}\left(Y\right)=\int_{0}^{\infty }\phi^{\prime }\left(u\right)F_{X}\left(u\right){\left[-\log F_{X}\left(u\right)\right]}^{q}du.$$

Making use of Lemma 1, the following theorem is immediately obtained.

**Theorem** **1.** *Let the condition in Lemma 1 hold. If $\phi^{\prime }\left(u\right)\geq 1\;\left(0\leq \phi^{\prime }\left(u\right)\leq 1\right),$ then ${\mathrm{CE}}_{q}\left(X\right)\leq \left(\geq \right){\mathrm{CE}}_{q}\left(Y\right)$ for all 0<q≤1.*

If ϕ(X)=aX+b with a>0 and b≥0, we have the following result:

(11) $${\mathrm{CE}}_{q}\left(aX+b\right)=a{\mathrm{CE}}_{q}\left(X\right),$$

for all 0<q≤1, which is given in Proposition 2.2 of Di Crescenzo et al. [24].

The r.h.s of (11) is not affected by *b* in the sense that the FCE is shift-independent.

The FCE of *X* can also be obtained in terms of the cumulative RHR function of *X* given in (4). The proof can be obtained from Proposition 2.1 of Di Crescenzo et al. [24].

**Corollary** **1.** *Let X have a finite FCE ${\mathrm{CE}}_{q}\left(X\right)$ for all 0<q≤1. Then:*

(12) $${\mathrm{CE}}_{q}\left(X\right)=E\left[T_{q}^{\left(2\right)}\left(X\right)\right],$$

*where:*

(13) $$T_{q}^{\left(2\right)}\left(x\right)=\int_{x}^{\infty }T^{q}\left(t\right)dt=\int_{x}^{\infty }{\left[\int_{t}^{\infty }\tau \left(u\right)du\right]}^{q}dt,\;x\geq 0.$$

We note that $T_{q}^{\left(2\right)}\left(x\right)$ in (13) is a decreasing convex function of x. This immediately allows us to obtain the following theorem.

**Theorem** **2.** *Let X have a finite mean μ=E(X). Then:*

$${\mathrm{CE}}_{q}\left(X\right)\geq T_{q}^{\left(2\right)}\left(\mu \right),$$

*for all 0<q≤1.*

**Proof.**  Noting that $T_{q}^{\left(2\right)}\left(x\right)$ is a convex function of *x*, the Jensen’s inequality is applicable in (12) and immediately provides the proof. □

For the definition of the decreasing convex order denoted by $X\leq_{dcx}Y$ and also the definition of the dispersive order denoted by $X\leq_{d}Y$, we refer the reader to Shaked and Shanthikumar [25]. In the following result, we demonstrate that the dcx order is a sufficient condition for ordering distributions according to their FCEs.

**Theorem** **3.** *If $X\leq_{dcx}Y$, then ${\mathrm{CE}}_{q}\left(X\right)\leq {\mathrm{CE}}_{q}\left(Y\right)$.*

**Proof.**  We first prove that if $X\leq_{dcx}Y$, then:

$$T_{q}^{\left(2\right)}\left(X\right)\leq_{dcx}T_{q}^{\left(2\right)}\left(Y\right),\;0<q\leq 1,$$

where the function $T_{q}^{\left(2\right)}\left(\cdot \right)$ is as given in (13). Since $T_{q}^{\left(2\right)}\left(\cdot \right)$ is a decreasing convex function for all 0<q≤1, it follows (see Section 4.A.1 of Shaked and Shanthikumar [25]) that $T_{q}^{\left(2\right)}\left(X\right)\leq_{dcx}T_{q}^{\left(2\right)}\left(Y\right)$. In particular, since the decreasing convex order implies the expectation ordering, thus ${\mathrm{CE}}_{q}\left(X\right)\leq {\mathrm{CE}}_{q}\left(Y\right)$. □

The following corollary follows from Theorem 3.1 of Di Crescenzo et al. [24], for which another proof is given here.

**Corollary** **2.** *If $X\leq_{d}Y$, then ${\mathrm{CE}}_{q}\left(X\right)\leq {\mathrm{CE}}_{q}\left(Y\right),$ for all 0<q≤1.*

**Proof.**  We remark that the dilation property implies $\phi^{\prime }\left(x\right)\geq 1.$ Using (10), the proof is obtained. □

Di Crescenzo and Longobardi [18] showed that the ordering distributions according to their associated cumulative entropies is not a result of ordering by the usual stochastic ordering of the underlying distributions. Similar results hold for the FCE.

## 3. Bounds and Inequalities

In what follows, we derive some upper and lower bounds for the FCE of nonnegative RVs. Let us first consider the following theorem.

**Theorem** **4.** *For all 0<q≤1:*

$${\mathrm{CE}}_{q}\left(X\right)\leq \pi_{q}\left(X\right),$$

*where $\pi_{q}\left(X\right)=\int_{0}^{\infty }{\bar{F}}^{q}\left(x\right)dx$*

**Proof.**  It is known that $x{\left(-\log x\right)}^{q}\leq {\left(1-x\right)}^{q}$ for all 0<x<1 and for every 0<q≤1. From (7), we achieve the result. □

**Remark** **2.** *We remark that $\pi_{q}\left(X\right)$ is known as the risk-adjusted premium introduced by Wang [26] lies in the framework of the proportional hazards model. It is remarkable that if $X_{1},X_{2},\ldots ,X_{n}$ are a random sample from F, then the minimum order statistic $X_{1:n}$ follows the proportional hazards model, which makes the results on this model useful in broader applications. If $X_{q}$ denotes a nonnegative RV with the survival function ${\bar{F}}_{q}\left(x\right)=P\left(X_{q}>x\right)$ for x≥0, then in the proportional hazards model we have:*

(14) $${\bar{F}}_{q}\left(x\right)={\left[\bar{F}\left(x\right)\right]}^{q},\;x\geq 0,\;0<q\leq 1,$$

*where $\bar{F}\left(x\right)$ is the survival function of the baseline model and q is the proportionality constant. For an insurer, the risk-adjusted premium automatically and consistently adjusts the risk burden relative to the expected loss for different risks. Moreover, it is additive when the risk is divided into layers, which makes it very attractive for insurance layer pricing. For detailed discussions, we refer the reader to Wang [26].*

The following theorem gives a sufficient condition for the FCE to be finite.

**Theorem** **5.** *If for some p>1/q, $E(X^{p})<\infty ,$ then ${\mathrm{CE}}_{q}\left(X\right)<\infty$ for all 0<q≤1.*

**Proof.**  Applying Theorem 4, we get:

$$\begin{array}{ccc} {\mathrm{CE}}_{q}\left(X\right) & \leq & \int_{0}^{\infty }{\bar{F}}^{q}\left(x\right)dx=\int_{0}^{1}{\bar{F}}^{q}\left(x\right)dx+\int_{1}^{\infty }{\bar{F}}^{q}\left(x\right)dx\\ & \leq & 1+\int_{1}^{\infty }{\bar{F}}^{q}\left(x\right)dx\leq 1+\int_{1}^{\infty }{\left[\frac{E(X^{p})}{x^{p}}\right]}^{q}dx\\ & = & 1+{\left[E\left(X^{p}\right)\right]}^{q}\int_{1}^{\infty }\frac{1}{x^{qp}}dx, \end{array}$$

where the third inequality is obtained by virtue of Markov inequality. The last integral is finite if $p>\frac{1}{q}$, and this completes the proof. □

A normalized version of cumulative residual entropy as well as cumulative entropy were studied in Rao [5] and Di Crescenzo and Longobardi [18]. We will now introduce a normalized version of the FCE. For a nonnegative RV *X* with finite nonvanishing risk-adjusted premium $\pi_{q}\left(X\right)$, we define the normalized FCE as:

(15) $${\mathrm{NCE}}_{q}\left(X\right)=\frac{{\mathrm{CE}}_{q}\left(X\right)}{\pi_{q}\left(X\right)}=\frac{1}{\pi_{q}\left(X\right)}\int_{0}^{\infty }F\left(x\right)T^{q}\left(x\right)dx,$$

for all 0<q≤1. Recalling Theorem 4, we anticipate that the normalized FCE takes values in [0,1]. For the special case q=1, we have the normalized cumulative entropy as:

$$\mathrm{NCE}\left(X\right)=\frac{\mathrm{CE}(X)}{E(X)},$$

which is discussed in Di Crescenzo and Longobardi [18].

In the next result, we discuss the relation of FCE with the CE by virtue of Jensen’s inequality. Indeed, it gives an upper bound for the FCE depending on the CE. Its proof is similar to that of the proof of Proposition 2 of Xiong et al. [23], and hence, we omit it.

**Theorem** **6.** *For X with the support [0,b] and 0<q≤1:*

$${\mathrm{CE}}_{q}\left(X\right)\leq b^{1-q}{\left[\mathrm{CE}\left(X\right)\right]}^{q}.$$

In the following, we show a lower bound of the FCE in terms of the differential entropy (1).

**Theorem** **7.** *For all 0<q≤1,*

$${\mathrm{CE}}_{q}\left(X\right)\geq C\left(q\right)e^{H(X)},$$

*where $C\left(q\right)=\exp\left(\int_{0}^{1}\log\left[x{\left(-\log x\right)}^{q}\right]dx\right)$ is a finite function of q.*

The proof of Theorem 7 is based on the log-sum inequality and is similar to Theorem 2 of Xiong et al. [23], where an analogous result is given for the FCRE. Another lower bound for the cumulative entropy is given below.

**Theorem** **8.** *For 0<q≤1:*

(16) $${\mathrm{CE}}_{q}\left(X\right)\geq \int_{0}^{\infty }F\left(x\right){\bar{F}}^{q}\left(x\right)dx.$$

**Proof.**  Recalling that $x{\left(-\log x\right)}^{q}\geq x{\left(1-x\right)}^{q}$ for 0<x<1 and 0<q≤1, from (7) we obtain the result. □

**Remark** **3.** *The right-hand side of (16) can be interpreted in probabilistic terms as follows:* *(i) In spirit of $\bar{F}\left(x\right)=1-F\left(x\right),$ the right-hand-side of (16) can be rewritten as:*

$$\int_{0}^{\infty }F\left(x\right){\bar{F}}^{q}\left(x\right)dx=\pi_{q}\left(X\right)-\pi_{q+1}\left(X\right),$$

*where $\pi_{q}\left(X\right)$ is the risk-adjusted premium.*

*(ii) Let X have a finite non-vanishing mean. Thanks to the use of Fubini’s theorem and recalling (14):*

$$\int_{0}^{\infty }F\left(x\right){\bar{F}}^{q}\left(x\right)dx=\int_{0}^{\infty }F\left(x\right){\bar{F}}_{q}\left(x\right)dx=\int_{0}^{\infty }f\left(t\right)\left[\int_{t}^{\infty }{\bar{F}}_{q}\left(x\right)dx\right]dt=E\left[X_{q}\right]E\left[{\bar{F}}_{q}^{e}\left(X\right)\right],$$

*where:*

$${\bar{F}}_{q}^{e}\left(t\right)=\frac{1}{E[X_{q}]}\int_{t}^{\infty }{\bar{F}}_{q}\left(x\right)dx,$$

*is the survival function of the ‘equilibrium variable’ of $X_{q}$ with survival function (14).*

*(iii) Since $\bar{F}\left(x\right)\leq {\bar{F}}^{q}\left(x\right),\;x>0,$ when 0<q≤1, we conclude that:*

$${\mathrm{CE}}_{q}\left(X\right)\geq \int_{0}^{\infty }F\left(x\right)\bar{F}\left(x\right)dx,$$

*being similar to that Proposition 4.3 of Di Crescenzo and Longobardi [15].*

The proportional reversed hazards rate (PRHR) model of an absolutely continuous nonnegative RV $X_{\theta }^{⋆}$ with proportionality constant θ>0 is defined by:

(17) $$F_{\theta }^{⋆}\left(x\right)={\left[F\left(x\right)\right]}^{\theta },\;\;x\geq 0.$$

It is worth mentioning that if $X_{1},X_{2},\ldots ,X_{n}$ are a random sample from *F*, then the maximum order statistic $X_{n:n}$ follows the PRHR model. One has $F\left(x\right)=e^{-T(x)},\;x\geq 0$, where T(x) is defined in (4). For more details on the applications and properties of PRHR model, see Di Crescenzo [27], Kirmani and Gupta [28], and references therein. In the forthcoming theorem, we give an upper bound for the FCE of $X_{\theta }^{⋆}$ depending on ${\mathrm{CE}}_{q}\left(X\right)$.

**Theorem** **9.** *For all 0<q≤1:*

$${\mathrm{CE}}_{q}\left(X_{\theta }^{⋆}\right)\leq \theta^{q}\;{\mathrm{CE}}_{q}\left(X\right),\;if\;\theta \geq 1,$$

*and the inequality is reversed whenever 0<θ≤1.*

**Proof.**  Recalling (7) and (17), we have:

$${\mathrm{CE}}_{q}\left(X_{\theta }^{⋆}\right)=\theta^{q}\int_{0}^{\infty }{\left[F\left(x\right)\right]}^{\theta }\;{\left[T\left(x\right)\right]}^{q}\;dx.$$

Since $F\left(x\right)\geq {\left[F\left(x\right)\right]}^{\theta },x\geq 0,$ when θ≥1, we obtain:

$${\mathrm{CE}}_{q}\left(X_{\theta }^{⋆}\right)\leq \theta^{q}{\mathrm{CE}}_{q}\left(X\right),$$

which completes the proof. For 0<θ≤1, we have $F\left(x\right)\leq {\left[F\left(x\right)\right]}^{\theta },x\geq 0,$ and hence, the desired result follows. □

By assuming that the proportionality constant θ>0 is integer, the following corollary follows from Theorem 9.

**Corollary** **3.** *Let $X_{1},\cdots ,X_{n}$ be i.i.d. random variables. Then:*

$${\mathrm{CE}}_{q}\left(\max\left\{X_{1},\cdots ,X_{n}\right\}\right)\leq n^{q}\;{\mathrm{CE}}_{q}\left(X_{1}\right),$$

*for all 0<q≤1.*

The next theorem is analogously established as Theorem 1 in Xiong et al. [23], and states that a sum of independent RVs are greater than that of either of them.

**Theorem** **10.** *For all 0<q≤1:*

$$\max\left\{{\mathrm{CE}}_{q}\left(Y\right),{\mathrm{CE}}_{q}\left(Y\right)\right\}\leq {\mathrm{CE}}_{q}\left(X+Y\right).$$

We now provide an expression for the FCE in terms of the mean inactivity time (MIT) function (cf. [29]). We recall that the MIT function of *X* which is defined by:

(18) $$\tilde{\mu }\left(t\right)=E\left[t-X|X\leq t\right]=\frac{1}{F(t)}\int_{0}^{t}F\left(x\right)\;dx,$$

for all t>0.

To this aim, we define the RV $X_{q}$ with the PDF as:

(19) $$f_{q}\left(x\right)=\frac{1}{\Gamma (q)}{\left[T\left(x\right)\right]}^{q-1}f\left(x\right),\;x\geq 0,$$

for all 0<q≤1, where T(x) is defined in (4).

**Theorem** **11.** *Let X have MIT function $\tilde{\mu }\left(x\right).$ Then, for all 0<q≤1:*

$${\mathrm{CE}}_{q}\left(X\right)=\Gamma \left(q+1\right)E\left[\tilde{\mu }\left(X_{q}\right)\right]$$

**Proof.**  It is clear that, for all 0<q≤1:

$$\int_{t}^{\infty }T^{q-1}\left(x\right)\tau \left(x\right)dx=\frac{T^{q}\left(t\right)}{q},\;t>0.$$

From the above relation and Equation (7), and using Fubini’s theorem, we obtain:

$$\begin{array}{ccc} E_{q}\left(X\right)\;\;\;\; & = & \;\;\;\;q\int_{0}^{\infty }F\left(t\right)\frac{T^{q}\left(t\right)}{q}\;dt=q\int_{0}^{\infty }F\left(t\right)\int_{t}^{\infty }T^{q-1}\left(x\right)\tau \left(x\right)\;dx\;dt,\\ & = & \;\;\;\;q\int_{0}^{\infty }T^{q-1}\left(x\right)\tau \left(x\right)\int_{0}^{x}F\left(t\right)\;dt\;dx\\ & = & q\Gamma \left(q\right)\int_{0}^{\infty }\frac{1}{\Gamma (q)}T^{q-1}\left(x\right)f\left(x\right)\tilde{\mu }\left(x\right)\;dx, \end{array}$$

where the last equality is obtained from (18). Thus, the results finally are obtained using (19) and the well-known relation Γ(q+1)=qΓ(q). □

**Example** **2.** *Let $X_{\left(n\right)}$ denote the maximum of a random sample of continuous nonnegative RVs $X_{1},\ldots ,X_{n}$, which are uniformly distributed on [0,1]. It is clear that the CDF of $X_{\left(n\right)}$ is $F_{\left(n\right)}\left(x\right)=x^{n}$ with the PDF $f_{n:n}\left(x\right)=nx^{n-1}$ for all 0<x≤1. From (18), we have ${\tilde{\mu }}_{\left(n\right)}\left(x\right)=\frac{x}{n+1}$ for 0≤x≤1. Thus, Theorem 11 implies that:*

$${\mathrm{CE}}_{q}\left(X_{n:n}\right)=\frac{\Gamma (q+1)}{n+1}E\left[X_{(n),q}\right]=\frac{n^{q}\Gamma \left(q+1\right)}{{\left(n+1\right)}^{q+1}},$$

*where the last equality is obtained by noting that:*

$$E\left[X_{(n),q}\right]={\left(\frac{n}{n+1}\right)}^{q},$$

*for all 0<q≤1.*

The following result is an interesting achievement.

**Theorem** **12.** *Let X have standard deviation σ(X) and FCE $E_{q}\left(X\right).$ Then:*

$$E_{q}\left(X\right)\leq q\sqrt{\Gamma (2q-1)}\sigma \left(X\right),$$

*for all 0.5≤q≤1.*

**Proof.**  For all 0<q≤1, by the Cauchy–Schwarz inequality, we obtain:

$$\begin{array}{ccc} {\left[\int_{0}^{\infty }\tilde{\mu }\left(x\right)T^{q-1}\left(x\right)f\left(x\right)dx\right]}^{2} & = & {\left[\int_{0}^{\infty }\tilde{\mu }\left(x\right)\sqrt{f(x)}\sqrt{f(x)}T^{q-1}\left(x\right)dx\right]}^{2}\\ & \leq & \left(\int_{0}^{\infty }{\tilde{\mu }}^{2}\left(x\right)f\left(x\right)dx\right)\left(\int_{0}^{\infty }T^{2q-2}\left(x\right)f\left(x\right)dx\right). \end{array}$$

Applying Theorem 21 of Toomaj and Di Crescenzo [30], it holds that:

$$\int_{0}^{\infty }{\tilde{\mu }}^{2}\left(x\right)f\left(x\right)dx=\sigma^{2}\left(X\right).$$

On the other hand, we have:

$$\int_{0}^{\infty }T^{2q-2}\left(x\right)f\left(x\right)dx=\Gamma \left(2q-1\right),$$

which is positive for all 0.5≤q≤1. Therefore, the proof is then completed. □

Through a proper expression, the FCE can be described differently. In fact, we provide an expression for the FCE based on the covariance of the proportional RHR function and the RV $T(X_{q}).$

**Theorem** **13.** *Let X have FCE ${\mathrm{CE}}_{q}\left(X\right).$ Then:*

$$Cov\left(X_{q},T\left(X_{q}\right)\right)=-\Gamma \left(q+1\right){\mathrm{CE}}_{q}\left(X\right),$$

*for all 0<q≤1.*

**Proof.**  First, from (19) it is verified that $E[T\left(X_{q}\right)]=q.$ Now:

$$\begin{array}{ccc} Cov(X_{q},T\left(X_{q}\right)) & = & E\left[X_{q}T\left(X_{q}\right)\right]-E\left[X_{q}\right]E\left[T\left(X_{q}\right)\right]\\ & = & E\left[X_{q}T\left(X_{q}\right)\right]-qE\left[X_{q}\right]. \end{array}$$

One can obtain:

$$E\left[X_{q}T\left(X_{q}\right)\right]=\int_{0}^{\infty }xT\left(x\right)f_{q}\left(x\right)dx=qE\left[X_{q+1}\right],$$

which implies that:

$$\begin{array}{ccc} E\left[X_{q}T\left(X_{q}\right)\right]-qE\left[X_{q}\right] & = & q\left(E\left[X_{q+1}\right]-E\left[X_{q}\right]\right)\\ & = & -q\Gamma \left(q\right){\mathrm{CE}}_{q}\left(X\right), \end{array}$$

where the last equality is obtained from Proposition 2.3 of Di Crescenzo et al. [24]. Therefore, we have the results using relation Γ(q+1)=qΓ(q). □

The study of waiting times for events is a topic of interest in many fields. To add the possibility of considering the inspection time *t* at which the system is found failed and modify the information of the updated distribution, a dynamic version of FCE can be considered. Let *X* be a lifetime under the condition that the system has failed prior to the time *t*. The distribution function of the inactivity time $X_{\left[t\right]}=\left[t-X|X\leq t\right],$ is given as:

$$F_{t}\left(x\right)=\left\{\begin{array}{ccc} \frac{F(x)}{F(t)}\; & \;\; & \;x\leq t\\ 1\; & \;\;\;\; & \;x>t. \end{array}\right.$$

The FCE for the inactivity time $X_{\left[t\right]}$ is:

$${\mathrm{CE}}_{q}\left(t\right)={\mathrm{CE}}_{q}\left(X;t\right)=\int_{0}^{t}\frac{F(x)}{F(t)}{\left[T(x)-T(t)\right]}^{q}dx,\;\;t>0,$$

for all 0<q≤1. As in Theorem 7:

$${\mathrm{CE}}_{q}\left(t\right)\geq C\left(q\right)e^{\bar{H}\left(t\right)},\;t>0.$$

where C(q) is given as in Theorem 7 and

$$\bar{H}\left(t\right)=-\int_{0}^{t}\frac{f(x)}{F(t)}\log\frac{f(x)}{F(t)}dx,\;t>0,$$

is the past entropy at time *t* of *X*; see Di Crescenzo and Longobardi [31] and Muliere et al. [32]. Moreover, using the FCE of inactivity time, Theorem 2.2 reformulated as:

$${\mathrm{CE}}_{q}\left(t\right)=\;E\left[T_{q}^{\left(2\right)}\left(X\right)\;|\;X\leq t\right],$$

where:

$$T_{q}^{\left(2\right)}\left(x;t\right)=\int_{x}^{t}{\left[-\log\frac{F(y)}{F(t)}\right]}^{q}\;dy,$$

for all t,x>0.

## 4. Characterization Properties

We produce a characterization property based on the maximum of a random sample. First, a technical lemma in the spirit of the Muntz–Szasz theorem (see Kamps [33]) is given.

**Lemma** **2.** *For any sequence of positive integers $\left\{n_{j},j\geq 1\right\}$, which is increasing in j, the sequence of polynomials $\left\{x^{n_{j}}\right\}$ is complete on L(0,1), iff:*

(20) $$\sum_{j=1}^{+\infty }n_{j}^{-1}=+\infty ,\;0<n_{1}<n_{2}<\cdots .$$

Let $X_{1},\cdots ,X_{n}$ be *n* i.i.d. RVs with PDF *f* and CDF *F*. We recall that the cumulative distribution function of the largest value of order statistics is $F_{n:n}\left(t\right)={\left[F\left(t\right)\right]}^{n},t\geq 0$. Now, we state the result.

**Theorem** **14.** *F and G belong to the same family of distributions, but for a change in location, iff:*

(21) $${\mathrm{CE}}_{q}\left(X_{n:n}\right)={\mathrm{CE}}_{q}\left(Y_{n:n}\right),$$

*for a fixed q and for all $n=n_{j},j\geq 1,$ such that $\sum_{j=1}^{\infty }n_{j}^{-1}$ is infinite.*

**Proof.**  The necessity is simple to prove. For the sufficiency part, if for two CDFs *F* and *G* Equation (21) holds, using the probability integral transformations U=F(X) and U=G(X), we haveL

(22) $$\int_{0}^{1}u^{n}{\left(-\log u\right)}^{q}\left[\frac{1}{f(F^{-1}\left(u\right))}-\frac{1}{g(G^{-1}\left(u\right))}\right]du=0.$$

If (22) holds for all $n=n_{j},j\geq 1,$ such that $\sum_{j=1}^{+\infty }n_{j}^{-1}=+\infty ,$ from Lemma 2, we derive that $f\left(F^{-1}\left(u\right)\right)=g\left(G^{-1}\left(u\right)\right)$ for all 0<u<1. It follows that $F^{-1}\left(u\right)=G^{-1}\left(u\right)+d,$ for all 0<u<1. This means that *F* and *G* belong to the same family of distributions, but for a location shift. □

In what follows, the completeness property of the characterization results of symmetric continuous distributions are applied through the FCE measures. We obtain a result for symmetric distributions based on the equality of the FCRE of the first-order statistic with the FCE of the last-order statistic. As usual, we denote by $X_{m:m}$ the maximum of a random sample having size *m* whose RVs are distributed as *X*. In a similar way, we denote by $X_{1:m}$ the minimum of a random sample having size *m* whose RVs are equal in distribution with *X*. The RVs $X_{1:m}$ and $X_{m:m}$ are known as the lifetimes of series and parallel systems in reliability engineering and there are many applications in this context (Barlow and Proschan [34]). By appealing to the techniques used in the proofs of Theorems 4 and 5 of Ahmadi and Fashandi [35] and further by applying Lemma 2, we acquire the following characterization results.

**Theorem** **15.** *Suppose $N=\{n_{j},j\geq 1\}$ is a sequence of positive integers which is strictly increasing such that (20) holds. Then, the following statements are equivalent:*

 *(i)*  *X has a symmetric distribution;*
 *(ii)*  *$E_{q}\left(X_{1:n}\right)={\mathrm{CE}}_{q}\left(X_{n:n}\right)$ for a fixed 0<q≤1 and for all n∈N.*

**Proof.**  The probability integral transformation, identified by U=F(X) provides that:

(23) $$\begin{array}{ccc} {\mathrm{CE}}_{q}\left(X_{n:n}\right) & = & n^{q}\int_{0}^{1}\frac{u^{n}{\left(-\log u\right)}^{q}}{f(F^{-1}\left(u\right))}\;du, \end{array}$$

(24) $$\begin{array}{ccc} E_{q}\left(X_{1:n}\right) & = & n^{q}\int_{0}^{1}\frac{u^{n}{\left(-\log u\right)}^{q}}{f(F^{-1}\left(1-u\right))}\;du, \end{array}$$

for all 0<q≤1. If *X* has a symmetric distribution, then by (23) and (24), we readily find that $E_{q}\left(X_{1:n}\right)={\mathrm{CE}}_{q}\left(X_{n:n}\right)$ for all 0<q≤1. For the sufficiency, by substituting Equations (23) and (24) into $E_{q}\left(X_{1:n}\right)={\mathrm{CE}}_{q}\left(X_{n:n}\right)$ yields:

(25) $$\int_{0}^{1}u^{n}{\left(-\log u\right)}^{q}\left(\frac{1}{f(F^{-1}\left(u\right))}-\frac{1}{f(F^{-1}\left(1-u\right))}\right)\;du=0.$$

Since, according to the hypothesis, Equation (25) holds for $n=n_{j},j\geq 1$, such that $\sum_{j=1}^{\infty }n_{j}^{-1}=\infty$, Lemma 2 implies:

$$f\left(F^{-1}\left(u\right)\right)-f\left(F^{-1}\left(1-u\right)\right)=0,\;a.e.\;u\in \left(0,1\right).$$

Thus, by Lemma 2, the proof is completed. □

An analogue theorem can be stated as Theorem 14 for the dynamic FCE.

**Theorem** **16.** *F and G belong to the same family of distributions, but for a change in location and scale, if and only if:*

$${\mathrm{CE}}_{q}\left(X_{n:n};t\right)={\mathrm{CE}}_{q}\left(Y_{n:n};t\right),$$

*for a fixed 0<q≤1, for all t≥0, and for all n∈N, where N is defined as in Theorem 15.*

**Proof.**  The necessity is obvious. Therefore, we prove the other part. For a fixed 0<q≤1 and for all n∈N if ${\mathrm{CE}}_{q}\left(X_{n:n};t\right)={\mathrm{CE}}_{q}\left(Y_{n:n};t\right),$ for all t≥0, then appealing to Theorem 14, one concludes that [X|X≤t] and [Y|Y≤t] follows the same distribution but for a change in location parameter, i.e., $f_{t}\left(x\right)=g_{t}\left(x+d\right),$x>0, for all t>0, where $f_{t}$ and $g_{t}$ are, respectively, used to represent the PDFs of [X|X≤t] and [Y|Y≤t]. Hence, $f\left(x\right)=\frac{F(t)}{G(t)}g\left(x+d\right)$, x>0 and this signifies that *F* and *G* lie within a same family of distributions, but with a change in the location and scale. □

## 5. Conclusions

We have considered an information measure closely related to the one recently presented by Di Crescenzo et al. [24]. This measure, called fractional CE, is an information measure based on cumulative entropy and the FE. The measure considered in this paper is a special case of the measure described in Di Crescenzo et al. [24], namely the fractional generalized cumulative entropy measure. We determined various thresholds for the FCE and also used the FCE to study the proportional RHR model, MIT function, standard deviation, and risk-adjusted premium. We also considered a dynamic version of the FCE and obtained some results related to this measure. Based on the identities between the amounts of FCE for maximum order statistics obtained from random samples, some characterization properties were presented. We also characterized a family of symmetric distributions based on the equality between the FCRE of the minimum order statistics of one random sample and the CE of the maximum order statistics of another random sample.

## Figures and Tables

**Table 1:** Fr*é*chet-Comparison between ${\mathrm{CE}}_{q}\left(X\right)$ and σ(X) for a>2 and 0.2<q≤1.

*q*	${\mathrm{CE}}_{q}\left(X\right)>\sigma \left(X\right)$	${\mathrm{CE}}_{q}\left(X\right)<\sigma \left(X\right)$
q=0.5	${\mathrm{CE}}_{0.5}\left(X\right)>\sigma \left(X\right)$ for a>3.696104	${\mathrm{CE}}_{0.5}\left(X\right)<\sigma \left(X\right)$ for 2<a<3.696104
q=0.6	${\mathrm{CE}}_{0.6}\left(X\right)>\sigma \left(X\right)$ for a>4.407095	${\mathrm{CE}}_{0.6}\left(X\right)<\sigma \left(X\right)$ for 2<a<4.407095
q=0.7	${\mathrm{CE}}_{0.7}\left(X\right)>\sigma \left(X\right)$ for a>5.107608	${\mathrm{CE}}_{0.7}\left(X\right)<\sigma \left(X\right)$ for 2<a<5.107608
q=0.8	${\mathrm{CE}}_{0.8}\left(X\right)>\sigma \left(X\right)$ for a>5.776157	${\mathrm{CE}}_{0.8}\left(X\right)<\sigma \left(X\right)$ for 2<a<5.776157
q=0.9	${\mathrm{CE}}_{0.9}\left(X\right)>\sigma \left(X\right)$ for a>6.395489	${\mathrm{CE}}_{0.9}\left(X\right)<\sigma \left(X\right)$ for 2<a<6.395489
q=1.0	${\mathrm{CE}}_{1.0}\left(X\right)>\sigma \left(X\right)$ for a>6.952219	${\mathrm{CE}}_{1.0}\left(X\right)<\sigma \left(X\right)$ for 2<a<6.952219

## Data Availability

No new data were created or analyzed in this study. Data sharing is not applicable to this article.
